# Laparoscopic treatment of deep endometriosis with a diode laser: our experience

**DOI:** 10.1007/s00404-021-06154-z

**Published:** 2021-08-26

**Authors:** Stefano Angioni, Luigi Nappi, Felice Sorrentino, Michele Peiretti, Angelos Daniilidis, Alessandro Pontis, Raffaele Tinelli, Maurizio Nicola D’Alterio

**Affiliations:** 1 Department of Surgical Sciences, Division of Gynecology and Obstetrics, University of Cagliari, Cagliari, Italy; 2 Department of Medical and Surgical Sciences, Institute of Obstetrics and Gynecology, University of Foggia, Foggia, Italy; 3 Department of Obstetrics and Gynecology, Hippokratio Hospital, Aristotle University of Thessaloniki, Thessaloniki, Greece; 4 Azienda Tutela Della Salute, Regione Sardegna, Cagliari, Italy; 5 U.O.C. Obstetrics and Gynecology, ‘Valle d’Itria’ Hospital, Martina Franca, Taranto, Italy

**Keywords:** Endometriosis, Deep endometriosis, Pelvic pain, Diode laser, Quality of life, Surgical treatment

## Abstract

**Purpose:**

To evaluate whether laparoscopic treatment with a diode laser is feasible, safe, and effective in symptomatic patients affected by deep endometriosis (DE).

**Methods:**

This retrospective study was performed using medical record data. The surgical reports, chronic pain scores, and quality of life (QoL) data were evaluated for 50 patients who had undergone laparoscopic surgery between November 2017 and March 2019 at two university hospitals (Monserrato (CA) and Foggia, Italy). Indications for surgery were chronic pelvic pain and/or infertility in patients who wished to conceive spontaneously. Endometriosis lesions/nodules were excised using a diode laser (Leonardo®, Biolitec® DUAL 45) that can combine 980 and 1470 nm wavelengths transmitted through a 1000 µm conical optical fibre.

**Results:**

The median patient age was 32 years (range 21–44), with a body mass index (BMI) mean of 21.7  ±  2.9 kg/m^2^. The mean operation time was 147 min (range 106–190). No intraoperative or early complications (< 30 days) were reported. All patients left the hospital, on average, within 3 days (range 2–9 days) after surgery. A significant improvement in pain was observed at the 3-, 6-, and 12-month follow-up (*p* < 0.01) in all patients. Moreover, patients reported a significant QoL improvement at the 12-month follow-up.

**Conclusion:**

The diode laser confirmed its feasibility and safety for treating endometriosis. During the shaving surgical procedure, the diode laser system ensures a safe and effective laparoscopic dissection of deep endometriotic lesions. Further comprehensive randomized trials are necessary to confirm these preliminary data in terms of efficacy, recurrence rates, and pregnancy outcomes.

## Introduction

Deep endometriosis (DE) is defined by the presence of endometriotic tissue over 5 mm in depth under the peritoneal surface [1]; it occurs in more than 20% of patients with endometriosis and often impairs fertility and quality of life (QoL) [2, 3]. As confirmed by recent studies, endometriosis has multiple etiological factors, with genesis in the field of genetics, immunology, endocrinology, and with undoubtedly environmental influences [4–8]. The presentation of DE often includes nodules that can affect the rectovaginal space, bowel, pelvic nerves, ureters, and/or bladder [9]; in rare conditions, DE may appear in other locations, such as the abdominal wall or thorax [10]. DE lesions can be single or multifocal and include symptoms such as dysmenorrhoea, chronic pelvic pain, dysuria, and dyschezia, in relation to the anatomical site involved [11]. Involvement of the full thickness of the intestinal wall is unusual, but endometriosis may deeply infiltrate the muscularis, inducing a constriction of the intestinal lumen and severe obstructive symptoms [12]. Many researchers agree that DE surgical excision can improve pain symptoms and quality of life and may also raise fertility chances [13–18].

For the treatment of DE, there is a wide variety of surgical procedures (especially for the removal of nodules from the bowel and ureter) using different devices; ultrasonic and plasma energy, cold scissors, and CO_2_ lasers, if handled by expert surgeons, ensure good results with low complication rates [9, 19, 20].

Laser (light amplification by stimulated emission of radiation) is a very useful energy source available for endoscopic surgery. The selective tissue absorption characteristics of different types of lasers can be used in surgery [21].

A neodymium:yttrium aluminum garnet (Nd:YAG) laser only has a 1064 nm wavelength and can be selectively absorbed by tissues containing haemoglobin, such as endometriotic tissue, with good haemostasis but poor properties in vaporization and cutting [21]. In the 1990s, the Nd:YAG laser was the first to be used for treating female pelvic pathologies (adhesions and endometriosis), but, due to its physical characteristics, Shirk et al. decided not to use it in the excision of deep endometriosis with bowel involvement [22].

A CO_2_ laser only has a 10,640 nm wavelength. It has high absorption in water and low absorption in haemoglobin; therefore, it is excellent for tissue cutting and vaporization but is not effective in tissue coagulation, especially when the tissue is wet with blood and physiological washing solution and therefore cannot be used in hysteroscopy [21]. Despite this effect, CO_2_ lasers have been used to treat women affected by DE in different settings. Meuleman et al. showed significant improvement in pain, sexual function, and quality of life in patients with DE and colorectal wall invasion with a good fertility rate and a low complication and recurrence rate after a CO_2_ laser laparoscopic radical excision combined with laparoscopic segmental bowel resection and re-anastomosis [20]. Kristensen et al. confirmed that endometriosis in the rectovaginal pouch and rectovaginal septum endometriosis could be effectively treated with CO_2_ laser laparoscopy with significant statistical differences between preoperative and postoperative pain scores, quality of life, frequency of sexual activity, and the percentage taking analgesics or non-steroidal anti-inflammatory drugs [23].

Over the years, lasers have also been used in other fields, such as in hysteroscopic surgery. In this field, different types of lasers have been used, for example, the Nd:YAG, potassium titanyl phosphate (KTP), and the argon laser [24].

**Fig. 1:**
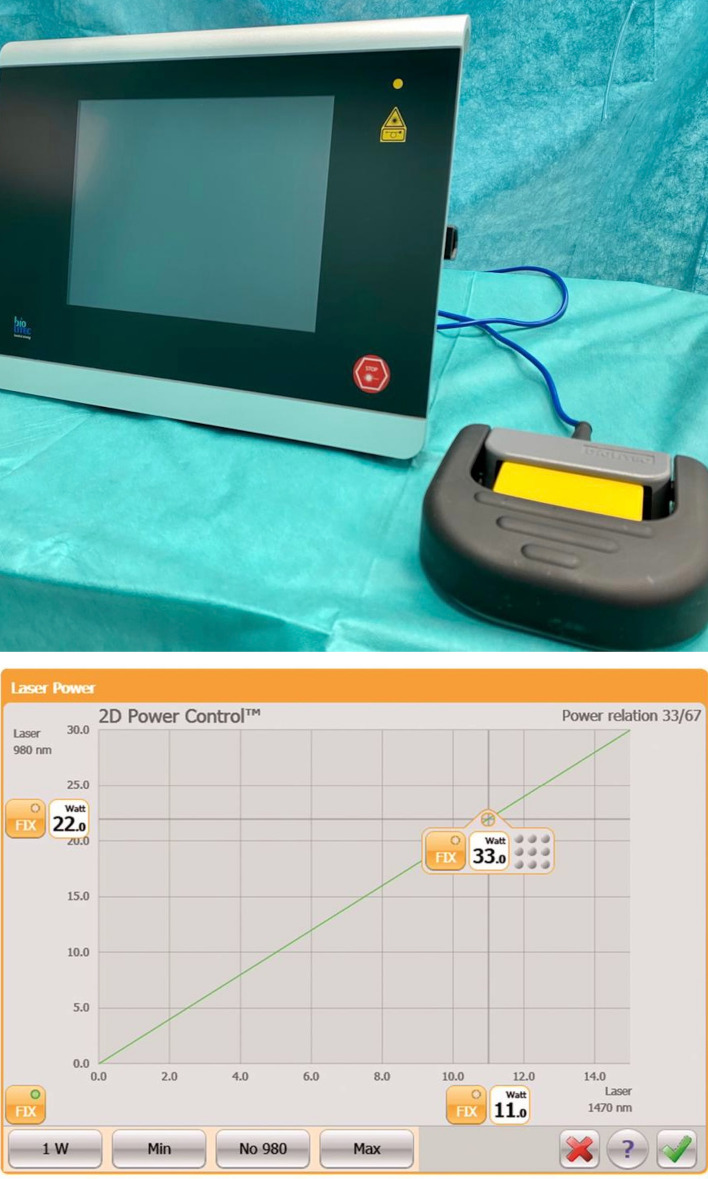
Leonardo DUAL 45 laser device

More recently, diode lasers have been introduced in endoscopic gynaecological surgery [25–30]. A diode is an electronic laser composed of two small semiconductor materials (50 μm each). The laser beam is produced by an electrical current flow across the diode, regulated by a microprocessor system and diffused to an optical fibre using an optical system. The optical fibre is the instrument that allows the light to reach the surgical site. The Leonardo® DUAL 45 diode laser (Biolitec®, Jena, Germany) generates two wavelengths, i.e. 980 and 1470 nm, that provide contemporary absorption in water (1470 nm) and haemoglobin (980 nm), with high performances in cutting, vaporizing, and haemostasis. The Leonardo® DUAL 45 diode laser is a device that can be easily used in laparoscopy and hysteroscopy, and is light and easy to handle; in addition, this laser device has two wavelengths, i.e. 1470 nm (mainly used for cutting) with a power from 0 to 15 Watt (W) and 980 nm (mainly used for coagulation) with a power from 0 to 30 W up to a resulting maximum of 45 W that can be obtained by mixing the two wavelengths (Fig. 1). The energy, measured in joules, is determined by the length of time this laser is operating at a specific power.

A comparison of the diode laser with the Nd–YAG laser shows that the thermal penetration of the diode laser is insignificant, thereby allowing a more precise and safer surgery even when close to delicate anatomical structures [25–29]. The aim of this study was to evaluate, using retrospective data, whether a laparoscopic treatment with a diode laser was feasible, safe, and effective in symptomatic patients affected by DE.

## Materials and methods

A double centre, retrospective, observational trial was approved by the Ethical Committee of the Azienda Ospedaliera Universitaria of Cagliari and included women who had undergone surgery with a diode laser (Leonardo® DUAL 45, Biolitec®, Jena, Germany) for DE at hospitals through the Divisions of Obstetrics and Gynecology of the University of Cagliari and the University of Foggia between November 2017 and March 2019. Patients were included if they were 18–45 years old at the time of surgery; had a laparoscopic diagnosis of DE with a histopathological examination; had undergone a laparoscopic surgery performed with a diode laser (Leonardo®, DUAL 45); had available a pre-treatment evaluation and preoperative and postoperative pain score and quality of life assessment; had a follow-up duration of at least 12 months from surgery.

We performed a retrospective search from a database of patients followed at the chronic pelvic pain and endometriosis specialist service of the Divisions of Obstetrics and Gynecology at the University of Cagliari and the University of Foggia. We reviewed the records of the patients involved in our study to collect data about preoperative, intraoperative, postoperative, and follow-up evaluation reports. The routine presurgical assessment consisted of the collection of the medical history data, physical and vaginal pelvic examination, pelvic ultrasound, and/or a magnetic resonance imaging scan. Among the preoperative data, the patient’s age, body mass index (BMI), previous abdominal surgery, indication for surgery, characteristics of DE lesions, and previous medical treatment were all assessed. Intraoperative parameters including overall operating time, blood loss, conversion rate, and complications were collected. Operative time was conventionally defined as the time from skin incision to skin closure. The estimated blood loss (EBL) was calculated by the difference in the total quantities of suctioned and irrigation fluids. All procedures performed during surgery were reported. Intraoperative complications were recorded based on a classification of intraoperative complications [31]. Postoperative parameters that were collected included postoperative pain, time to discharge, and early complications (within 30 days of the procedure) and late complications (> 30 days) measured according to the Clavien–Dindo classification of surgical complications scale [32]. Systematic postoperative clinical and symptomatic assessments were achieved at 3 months, 6 months, and 1 year. At each follow-up visit, a full evaluation was conducted that consisted of a patient interview to define subjective symptoms, administration of a validated questionnaire, as well as a gynaecological pelvic investigation and a transvaginal ultrasound evaluation. The primary outcome measures were symptom outcomes; the secondary outcome measures were recurrence, reoperation rate, and complications. Both centres routinely evaluate pain scores and quality of life through validated questionnaires before and during the follow-up of the patients that undergo surgical treatment for chronic pain. Pain level was assessed with the modified Biberoglu and Behrman (B and B) symptom scale [33, 34]. In each of the five categories of the B and B symptom scale (dysmenorrhoea, dyspareunia, pelvic pain, tenderness and induration on palpation), symptoms and signs range on a scale from 0 (no discomfort) to 3 (severe symptoms). Moreover, for the patients attending our centres, their quality of life and health-related gratification were routinely measured with the Medical Outcomes Survey Short Form 36 (SF-36), which is the most fully applied common instrument for evaluating health-related quality of life [35].

All surgeries were performed by two skilled surgeons (S.A. and L.N.). Patients received antimicrobial prophylaxis before surgery. A surgical approach was performed to achieve a complete DE excision following the conventional steps and surgical procedures previously described [34]. In all cases, we used a diode laser with a 1000 μm conical fibre with an average power of 15 W (Leonardo®, DUAL 45), mixing the two wavelengths of 980 nm and 1470 nm) inside a 5 mm sheath to perform the cutting, coagulation, and dissection. The importance of using two wavelengths is that you can choose when you need more cutting or haemostasis. For example, to limit damage to the urinary tract during the removal of a ureteral nodule, for the dissection of the same, the surgeon can choose to use a power from 0 to 15 W for cutting only (1470 nm wavelength), or instantly combine with the other wavelength (980 nm) when there is bleeding and select the depth at which they want the laser to act, based on a power (from 0 to 30 W). In particular, the laser fibre was used to perform the isolation and excision of endometriosis nodules from the bowel, ureter, bladder, and retroperitoneal tissues.

### Statistical analysis

Data were analysed with IBM SPSS Statistics 25.0. The analysis of data included the patients’ ages, surgical procedures, operating time, intraoperative and postoperative complications, and time to discharge. The results were reassumed as the mean and standard deviation for continuous data and as the frequency for categorical data. The Wilcoxon matched-pairs test for continuous variables was used to assess the intergroup variations between baseline and follow-up values. We used Fisher’s exact test to compare qualitative variables. A value of *p* < 0.05 was estimated to be statistically significant.

## Results

Between November 2017 and March 2019, 210 patients underwent surgical treatment for endometriosis at the two centres. In total, 50 of these patients who met the inclusion criteria and did not fall under any exclusion criterion were scheduled to undergo laparoscopic DE excision with a diode laser and had presurgical, surgical, and follow-up data available. All detectable endometriotic implants underwent radical laparoscopic excision. In all cases, a definitive diagnosis of DE was histologically confirmed by finding stroma and endometrial glands in the excised tissue. The median patient age was 32 years (range 21–44), and the mean BMI was 21.7  ±  2.9 kg/m^2^.

The preoperative clinical characteristics of patients are shown in Table 1. All the patients presented with chronic pain and had not responded to medical treatment for the pain; 30 patients (60%) had undergone prior abdominal surgery and 20 patients (40%) experienced infertility with the desire to conceive spontaneously. Preoperative questionnaires regarding symptoms indicated the presence of severe dysmenorrhoea in 50 patients (100%), severe dyspareunia in 44 patients (88%), severe chronic pelvic pain in 50 patients (100%), dyschezia in 20 patients (40%), and dysuria in 10 patients (20%).

**Table 1:** Preoperative characteristics of the patients

	All patients (*n* = 50)
Age (years), median (interval)	32 (21–44)
Body mass index, kg/m^2^ mean ± SD	21.7 ± 2.9
Indication for surgery, *n* (%)	
Chronic pelvic pain	50 (100)
Dysmenorrhoea	50 (100)
Dyspareunia	44 (88)
Dyschezia	20 (40)
Dysuria	10 (20)
Infertility	20 (40)
Prior abdominal surgery, *n* (%)	30 (60)
Prior medical treatment, *n* (%)	50 (100)

**Fig. 2:**
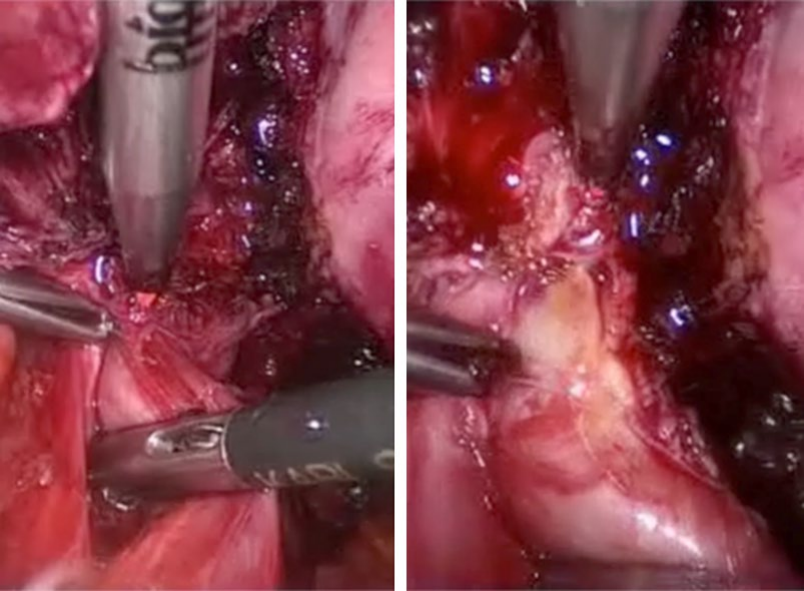
Diode laser ureterolysis

**Fig. 3:**
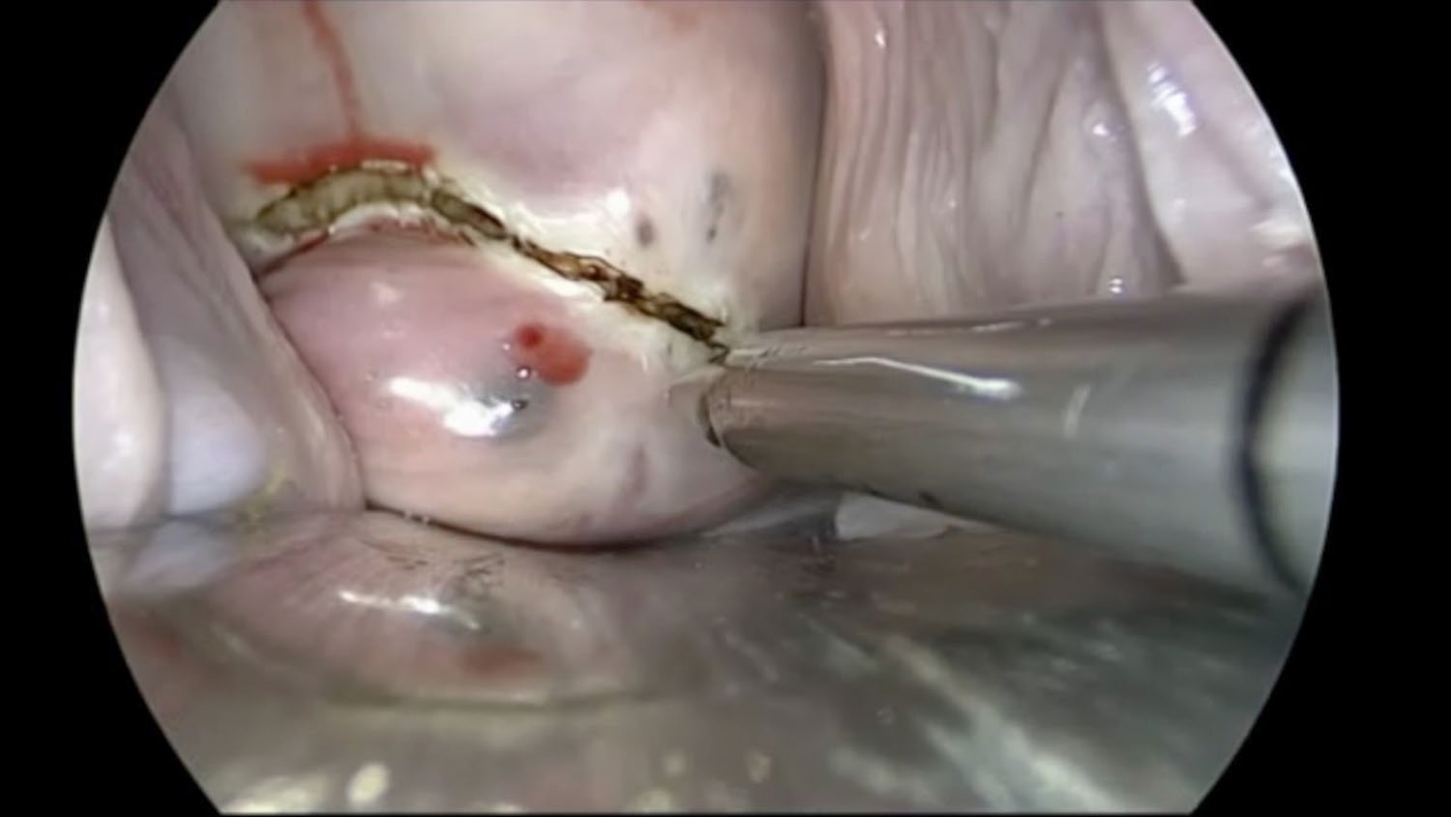
Vaginal nodule delimitation with the diode laser by vaginal route

**Fig. 4:**
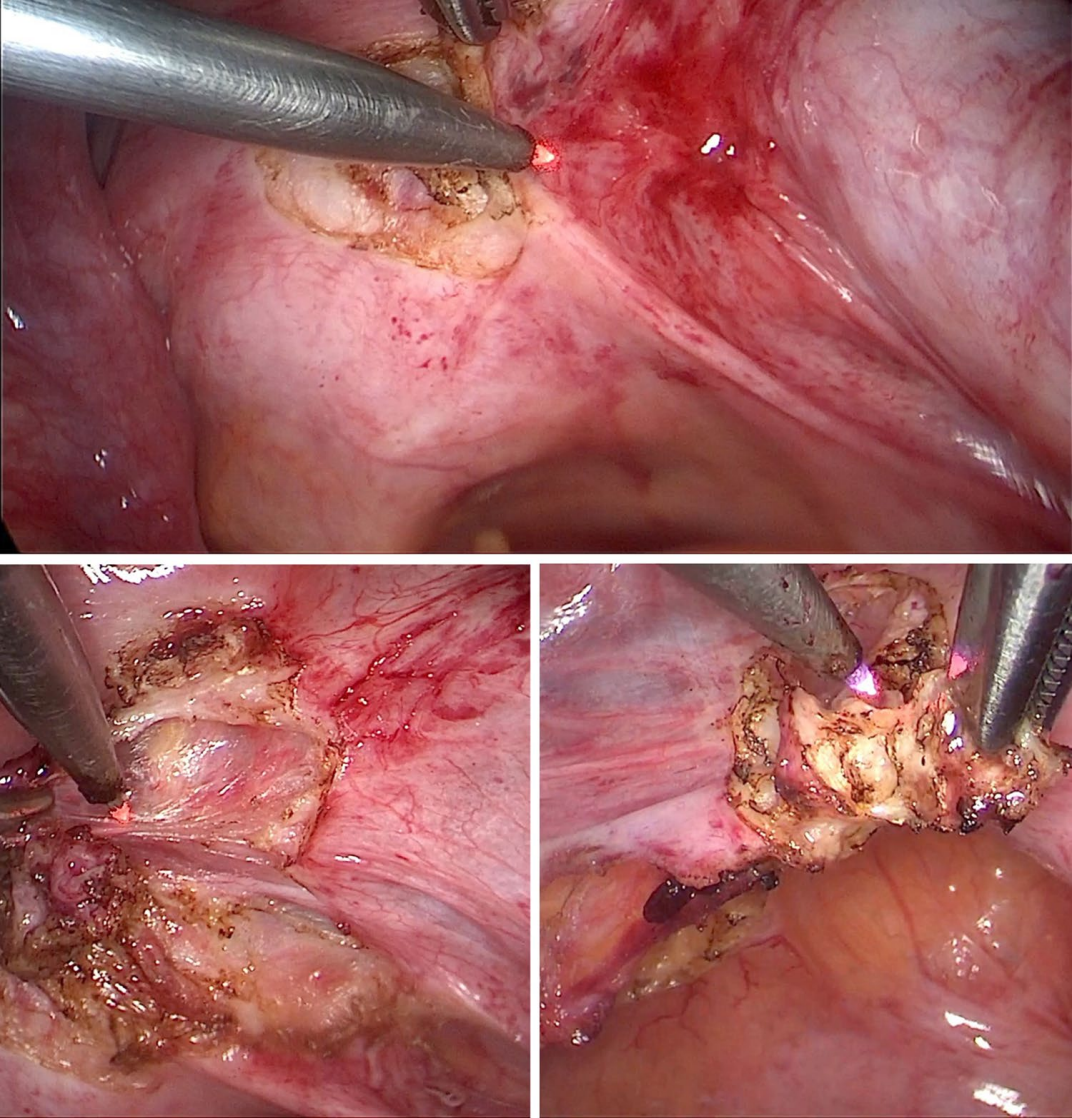
Laparoscopic excision of deep endometriosis infiltrating uterosacral ligaments

The surgical procedures performed using a diode laser fibre were adhesiolysis, ureterolysis **(**Fig. 2), posterior fornix resection with laser delimitation of the nodule by vaginal route (Fig. 3), excision of DE infiltrating the uterosacral ligaments (without their resection) (Fig. 4), bowel shaving (superficial peeling of bowel serosal and subserosal endometriosis) [36], full-thickness anterior rectal wall excision (selective excision of the bowel endometriotic lesion without opening of the bowel wall) [36], rectosigmoid resection, and partial bladder resection. In ten cases, the bladder nodule was bordered with a laser transurethral approach before complete excision by laparoscopy, as previously described [37]. Table 2 shows the numbers and percentages of the surgical findings. The mean operative time was 147 min (range 106–190). All the procedures were completed laparoscopically, and no conversion to laparotomy was required. The estimated blood loss was 129.2  ±  46.8 ml. Neither intraoperative nor early or late complications were reported. All patients left the hospital, on average, within 3 days (range 2–9 days) after surgery. Four patients had a postoperative fever of > 38 °C, which decreased after 2 days of antibiotic treatment. Thirty-five (70%) of the 50 patients were free of analgesic drugs on Day 2.

**Table 2:** Surgical findings

	All patients (*n* = 50)
Surgical procedures, *n* (%)	
Adhesiolysis	50 (100)
Posterior fornix resection	30 (60)
Excision of DE infiltrating uterosacral ligaments	35 (70)
Ureterolysis	40 (80)
Partial bladder resection	10 (20)
Bowel shaving	35 (70)
Full-thickness anterior rectal wall excision	5 (10)
Rectosigmoid resection	6 (12)
Mean operative time (min), mean (interval)	147 (106–190)
Intraoperative complications, *n* (%)	0 (0)
Conversion to laparotomy, *n* (%)	0 (0)
Estimated blood loss, ml mean ± SD	129.2 ± 46.8
Hospital stay (days), mean (interval)	3 (2–9)
Number of patients with fever > 38 °C, *n* (%)	4 (8)
Number of patients analgesic-free at day 2, *n* (%)	35 (70)
Hormonal therapy after surgery (dienogest 2 mg), *n* (%)	30 (60)
Postoperative pregnancy intent, *n* (%)	20 (40)

**Fig. 5:**
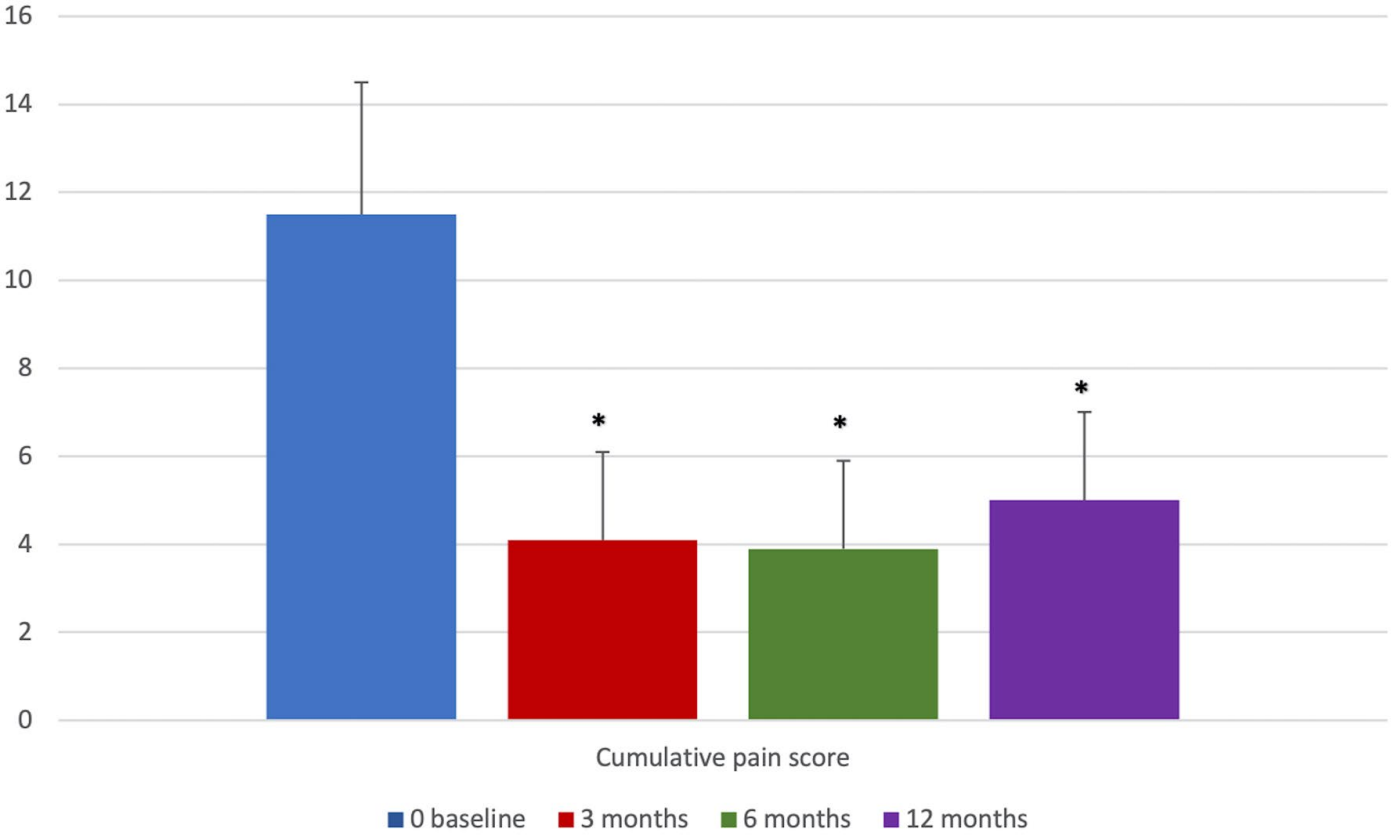
Cumulative pain scores at 0, 3, 6, and 12 months (*p < 0.01 vs. baseline)

**Fig. 6:**
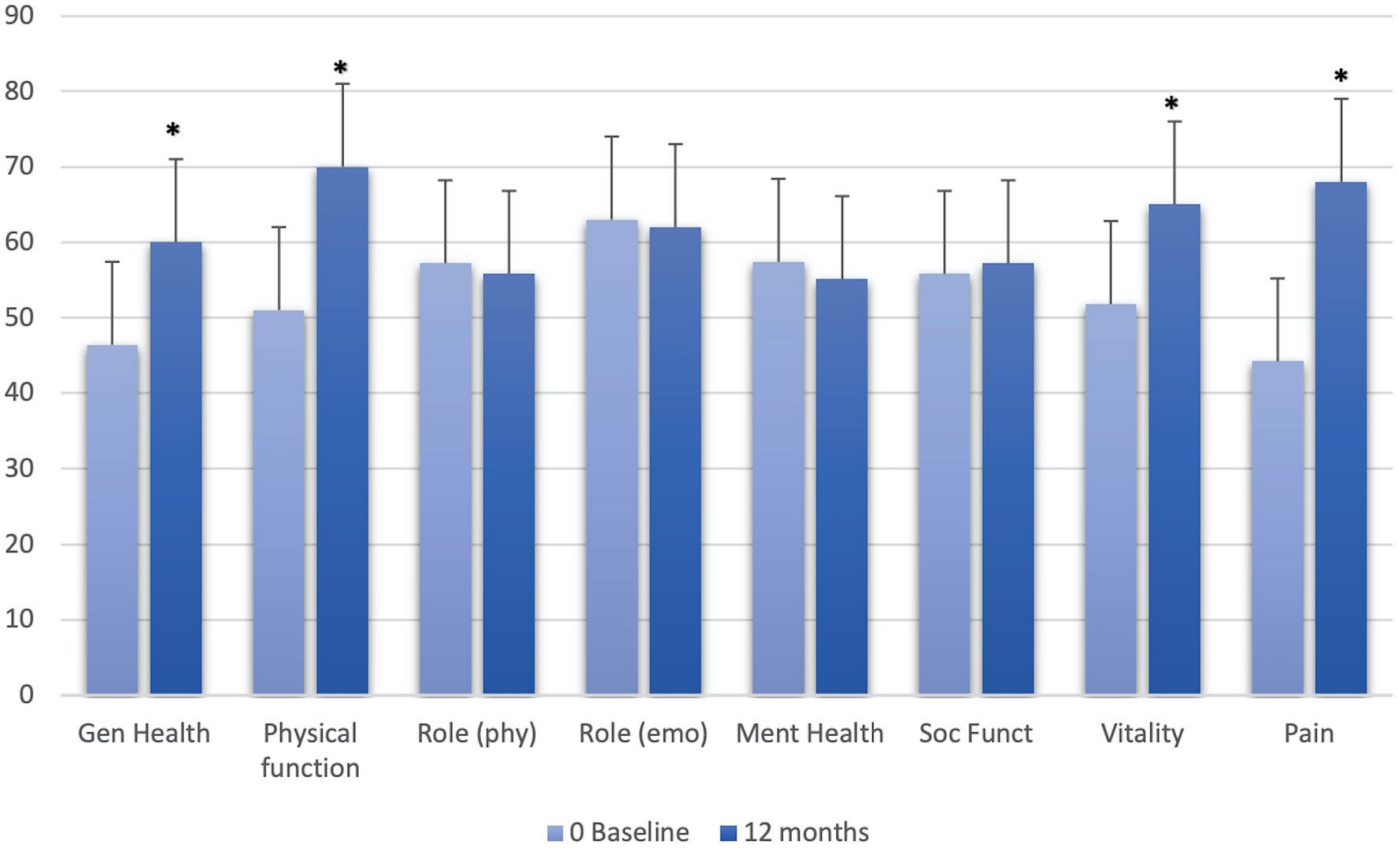
Differences in the patients’ quality of life, as assessed by SF36, before surgery and at 12 months follow-up (*p < 0.01 vs. baseline)

A statistically significant improvement in cumulative pain scores was observed at 3, 6, and 12 months of follow-up (*p* < 0.01) (Fig. 5). Moreover, at 1-year follow-up, patients treated showed significant improvement (*p* < 0.01) in four domains of the SF-36 questionnaire, i.e. physical function, general health, pain and vitality as compared with the baseline (Fig. 6). Thirty patients did not take any hormonal treatment after surgery due to a desire for pregnancy. Twenty patients began continuous administration of dienogest (2 mg per day) at discharge to prevent clinical and symptom recurrences.

## Discussion

Lasers are instruments that release coherent light (spatially and temporally) using an optical amplification system based on stimulated discharge of electromagnetic radiation. Laser beams can concentrate high power in a minimal area and have been widely used in medicine and surgery since the 1990s. Nanoscience has created a diode laser for a new generation of medical devices that combines a small-sized device with lower cost than older laser devices [38]. The Leonardo® laser by Biolitec®, in particular, can use two different wavelengths (980 nm and 1470 nm) emitted simultaneously through optical fibres to give a contemporaneous absorption of haemoglobin and water. Good results using a diode laser have previously been demonstrated in hysteroscopic polypectomy and metroplasty, which have included extreme precision of cutting, precise and constant control of tissue vaporization in the complete absence of bleeding, controlled power of penetration/deepening, a high capacity of haemostasis, the absence of electrical interferences, high safety, and good tolerance by the patients [28, 29]. In our previous laparoscopic reports, we have demonstrated that the diode laser DWLS has limited thermal dispersion, with precise tissue cutting and haemostasis, while performing prophylactic salpingectomy and bilateral salpingo-oophorectomy, avoiding fallopian tube histology distortion, therefore, improving the prognosis of breast cancer gene (BRCA) carriers [25, 26]. Moreover, the diode laser DWLS has also previously demonstrated low thermal penetration (with good results on fertility-sparing parameters such as antral follicular counts, anti-Mullerian hormone, and ovarian volume), when haemostasis control is needed after endometrioma stripping, and therefore it is a safe and accurate surgery for delicate anatomical structures such as the ovarian hilum [27]. As can be observed from the results of this study, the diode laser DWLS, due to its precise surgical cutting characteristics and low heat dispersion, can be used for safe surgical interventions on delicate anatomical systems, such as ureters and the bowel, that are often involved in case of DE.

Although there are still no definitive guidelines from any of the relevant societies as to when surgery should be suggested, or for the specific types of interventions to be performed for DE [39], many studies have acknowledged that pain and quality of life are improved by the surgical resection of DE [14–16]. Important complications (both minor, 1.1%, and major, 3.9%) are correlated with DE surgical resection, especially when the bowel is affected (minor, 1.0%, and major, 6.3%) [39]. Surgical treatment of DE is indicated for those women who decline or have contraindications to hormone therapy, as well as for patients who experienced a failure or incomplete relief of symptoms after medical treatment [39, 40]. The surgical approaches are different, depending on the experience of the surgeon and the acceptance by the patient of possible severe complications [9, 32, 41]. In general, the consensus among surgeons is that all visible lesions should be removed for prolonged improvement of symptoms. In addition, a specialized surgery should be taken into account to preserve organ functions, to reduce complications, and for nerve sparing [42–45]. One characteristic of a conic dual length diode laser is that the optical fibre of 1 mm can be easily handled through a 5 mm sheath, suggesting that a diode laser could be used in the treatment of DE. We successfully performed the surgeries in 50 patients without any early or late complications. The ability of this laser to act in air and fluids allowed us to border a large bladder nodule in cystoscopy with a Bettocchi® hysteroscope (Karl Storz®, Tubingen, Germany). It also allowed us to obtain a secure excision in laparoscopy, while avoiding any possible damage to the trigonus, for selective removal of the bladder wall involved with the disease, as previously described with other devices [37]. In previous cases of bowel endometriosis and nodules < 3 cm, we usually performed the excision with cold scissors. In our opinion, small/mid-rectal nodules that penetrate exclusively into the muscular layer and are free of rectal lumen advanced stenosis can be entirely excised without opening the bowel. The major benefit of rectal shaving and full-thickness anterior rectal wall excision is the possibility of treating a bowel nodule without opening and suturing the rectal wall. This surgery is not easy, and cold scissors can induce bleeding. In addition, sometimes, the cutting control is not very good. This surgical step was greatly facilitated by the use of the laser in the 40 cases in which we performed the procedure. Moreover, in six other cases with bigger nodules and important bowel stenosis, we performed a bowel resection. In these cases, all the steps before the sectioning of the bowel with staplers (development of retroperitoneal spaces, isolation of the ureters and their shaving, and removal of all other visible nodules) were accomplished with the laser.

In our study, 20 women also had associated infertility. Few data are available on the reproductive outcomes following DE surgical procedures. In subfertile women with stage III/IV endometriosis r-ARSM, no controlled trials have been performed to compare the reproductive outcomes after surgery and after a suitable period of watchful waiting. Prospective cohort studies have reported a higher rate of spontaneous pregnancy after laparoscopic surgery than watchful waiting [46]. In their prospective cohort study, Bianchi et al. attempted to compare the IVF outcomes in women with DE-associated infertility who underwent laparoscopic excision of endometriosis before IVF to those in patients who did not undergo surgery before IVF. The probabilities of becoming pregnant were 2.45 times higher in the surgical treatment group before IVF [17]. The impact of surgery appears to be controversial in this field. The current studies are observational and not strong enough to consider any definitive conclusions. Although some observational studies have concluded that endometriosis surgical treatment may increase pregnancy rates, ovarian damage may occur after the surgery, leading to a reduction in the number of antral follicles [47].

It is our practice to suggest postoperative medical therapy to reduce the risk of recurrence, excluding patients seeking pregnancy. In fact, some studies have demonstrated decreased pain and less recurrence of endometriosis lesions in women with postoperative amenorrhoea [48, 49]. The evaluation of pain and quality of life scores before and after surgery verified the efficacy of the surgical approach and confirmed the preliminary results of our pilot study [50].

This study had several limitations. One is the absence of a control group. It was also a retrospective analysis with only a small number of patients. The extensive laparoscopic experience of the surgeons (S.A. and L.N. have more than 25 years of experience) does not allow the drawing of definitive conclusions. Another limitation of this study was the short length of follow-up (1 year) and the use of medical treatment after surgery in 20 patients. It is clear that the rate of clinical recurrences increases with an increase in the follow-up period and that medical treatment may delay this problem. Nevertheless, all treated patients had not responded to medical treatment before surgery, and a significant improvement of pain and QoL was recorded in the follow-up. Indeed, diode laser surgery is a new technology, and our study is the first report on its use in a case series of women with DE. We believe that two-arm studies comparing diode surgery to other techniques and randomized clinical trials are still needed.

## Conclusion

Our study demonstrates that a diode laser could be a useful device for the laparoscopic treatment of DE. Its extreme cutting precision and controlled power penetration depth, combined with the good haemostatic capacity and high safety, makes it a promising new instrument, in particular, for shaving and excisional procedures of the bowel and the ureters. The improvement in symptoms and quality of life of our treated patients supports the efficacy of the approach, even if more studies are needed.

## Data Availability

The datasets used and/or analysed during the current study are available from the corresponding author on reasonable request.
